# High electrocatalytic activity of Pt on porous Nb-doped TiO_2_ nanoparticles prepared by aerosol-assisted self-assembly

**DOI:** 10.1039/d2ra03821h

**Published:** 2022-08-10

**Authors:** Xin Fu, Ruisong Li, Yucang Zhang

**Affiliations:** College of Ocean Food and Biological Engineering, Jimei University Xiamen 361021 China yczhang@jmu.edu.cn; Key Laboratory of Advanced Materials of Tropical Island Resources, Ministry of Education, School of Chemical Engineering and Technology, Hainan University Haikou 570228 China

## Abstract

This study explores an aerosol-assisted method to prepare an efficient support for the Pt catalyst of polymer electrolyte membrane fuel cells (PEMFCs). Titania nanoparticles and mesoporous niobium-doped titania nanoparticles were prepared by aerosol-assisted self-assembly using titanium(iv) isopropoxide and niobium(v) ethoxide as the titanium and niobium sources for application as non-carbon supports for the platinum electrocatalyst. The structural characteristics and electrochemical properties of the supports were investigated by transmission electron microscopy, X-ray diffraction, Fourier-transform infrared spectroscopy, X-ray photoelectron spectroscopy, electron paramagnetic resonance, inductively coupled plasma optical emission spectrometry, and dynamic light scattering. The Brunauer–Emmett–Teller method was used to calculate the specific surface areas of the samples, and the pore size distribution was also examined. The results demonstrated that under a radial concentration gradient, the aerosol droplets self-assembled into a spherical shape, and mesoporous supports were obtained after subsequent removal of the surfactant cetyltrimethylammonium bromide by annealing and washing. The hydrothermal technique was then used to deposit platinum on the TiO_2_-based supports. The electrical conductivity of the non-carbon support was enhanced by the strong metal–support interaction effect between the platinum catalyst particles and the porous niobium-doped TiO_2_ support. The half-wave potential, electrochemical surface area, mass activity, and specific activity of the obtained Pt/Nb-TiO_2_ catalyst all surpassed those of commercial Pt/C.

## Abstract 2

A niobium-doped titanium dioxide electrocatalyst support for proton-exchange membrane fuel cells was prepared by an aerosol-assisted method and then loaded with platinum nanoparticles in the presence of ethylene glycol as a reducing agent.

## Graphical abstract

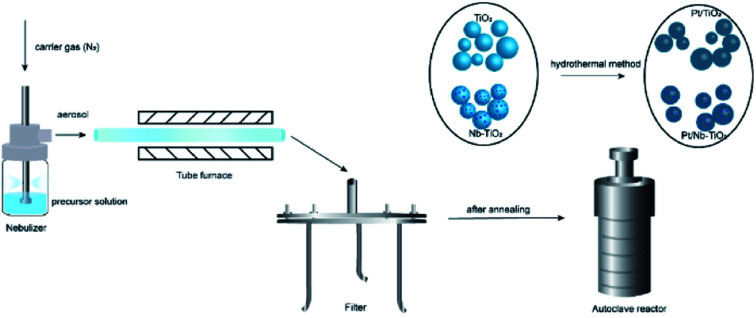

## 1. Introduction

To fulfill the growing global demand for high-density energy production and storage in the context of the eventual depletion of fossil fuels, researchers are attempting to develop highly efficient, environmentally friendly, renewable, and clean alternative energy sources.^1^ Proton-exchange membrane fuel cells (PEMFCs) are commonly regarded as a viable option for long-term energy conversion. Because of their high energy conversion, zero carbon emissions, and low operating temperature, PEMFCs are considered to be ideal renewable energy-storage systems for overcoming future energy concerns.^2,3^ However, they require remarkably active and stable electrocatalysts for both fuel oxidation and the oxygen reduction reaction (ORR).^4^ The most frequently used ORR electrocatalysts in PEMFCs are composed of platinum nanoparticles supported on porous carbon materials (*e.g.*, graphene oxide, carbon nanotubes, mesoporous carbon, and nitrogen-doped graphene). Although these carbon-based supports have the advantages of low cost and good electrical conductivity, they are also susceptible to corrosion and have low durability.^5^ Consequently, replacing carbon-based supports with materials displaying excellent electrical conductivity and corrosion resistance under typical PEMFC operating conditions is crucial for improving system longevity.^6^ Transition-metal oxides are interesting candidates for support materials because they can exhibit the strong metal–support interaction (SMSI) effect with platinum. The SMSI effect describes variations in the chemisorption behavior of organic molecules on metals, where supports of variable oxidation states affect metal adhesion properties to alter the electronic structure of the metal.^7^ The strong adsorption properties gained by changing the electronic structure of the metal have been reported to account for the increased catalytic stability and activity.^8,9^

Because of their extraordinary mechanical strength and chemical stability in extremely acidic or basic environments, metal oxides represent appealing support materials for electrode catalysts.^10^ Conductive or semi-conductive metal oxides such as Ti_0.7_Mo_0.3_O_2_, titanium–ruthenium oxide, Ta_0.3_Ti_0.7_O_2_, oxygen-deficient titanium dioxide (TiO_2_), indium tin oxide, antimony tin oxide, and TiO_2_/C have been investigated as suitable support materials.^11–14^ Because metal oxides and platinum nanoparticles have such a strong surface contact, the application of the former as support materials is expected to improve the stability and corrosion resistance of platinum catalysts. On account of its stability under typical fuel-cell operating conditions, low cost, commercial availability, lack of toxicity, and amenability to shape and structural alterations, TiO_2_ is regarded as a unique support material among these metal oxides.^15^ TiO_2_ is a well-known semiconductor with good stability and high abundance on earth. As a result, TiO_2_ has the potential to be used in energy conversion and storage systems.^16^ However, the sluggish charge mobility of TiO_2_ greatly reduces its overall performance. Niobium ion (Nb^5+^) is frequently used as the ideal dopant to modify the electrical characteristics of TiO_2_ in order to enhance electron transport. The Nb-doping presented two crucial justifications: (1) niobium(v) ethoxide has a 3.4 eV bandgap, which is very close to the TiO_2_ bandgap of 3.2 eV; (2) its comparable ionic radius (64 pm) to that of titanium (60.5 pm).

In previous studies, a variety of methods have been used to synthesize Nb-TiO_2_, including hydrothermal, sol–gel, ionic liquid microemulsion aided synthesis, and template-assisted multistep synthesis approaches.^17–19^ However, these methods generally suffer from low product yields and comprise multiple steps, potentially limiting reproducibility. Owing to their practicality, scalability, cost effectiveness, and minimal waste generation, aerosol-assisted techniques for producing hollow spherical nanoparticles have gained favor in recent years. The obtained nanomaterials possess a large number of active sites owing to their high specific surface area, homogeneous pore size, and large pore volume.^20^ These methods usually result in a range of nanostructured composites, which combine nanocrystalline host materials with carbon frameworks to drastically improve electrochemical performance. Guan *et al.* combined an aerosol-assisted process with the hot-press technique to establish a scalable route to a bio-inspired synthetic nanocomposite.^21^ In addition, Meng *et al.* devised an aerosol-assisted hydrothermal process for synthesizing zeolites, particularly Sn-beta zeolites.^22^ Furthermore, Poostforooshan *et al.* described a simple new method for preparing hollow mesoporous silica microspheres by spray-drying colloidal silica nanoparticles and polymethacrylate-based copolymer/surfactant nanosphere templates followed by calcination.^23^

In this work, in response to these eye-opening reports concerning TiO_2_ catalytic supports and the application of aerosol-assisted techniques,^24^ hollow mesoporous TiO_2_ nanoparticles with a large specific surface area were generated by aerosol-assisted self-assembly followed by annealing. As a synthetic technique, this gas-phase technology has several advantages over more conventional liquid-phase approaches, including simple operation, rapid particle formation, and the ability to continuously produce spherical particles.^25^ Because the dried samples departing the furnace were collected using membrane filters, no time-consuming separation procedures were necessary. Surprisingly, the aggregation of the formed nanoparticles could be minimized because each droplet can be regarded as an independent microreactor while passing through the furnace following atomization.^26^ These nanoparticles were then applied as a support for a platinum electrocatalyst. In addition, a niobium-doped TiO_2_ support with high electrical conductivity was prepared by a similar approach. The titanium and niobium sources were combined in a precursor solution containing cetyltrimethylammonium bromide (CTAB), and after the aerosol-assisted self-assembly the platinum was loaded by a hydrothermal process using ethylene glycol as a reducing agent. Finally, the electrocatalytic performance of the samples was evaluated.

## 2. Experimental

### 2.1. Catalyst preparation

#### 2.1.1. Synthesis of TiO_2_ and Nb-TiO_2_ nanoparticle supports

TiO_2_ nanoparticles were synthesized by aerosol-assisted self-assembly. CTAB (2 g, Aladdin) was dissolved in ethanol (50 mL) followed by the addition of titanium(iv) isopropoxide (TTIP; 7.1 g, 0.954 g cm^−3^, Macklin), and the resulting mixture was subjected to ultrasonication for 10 min. Acetic acid solution (1.0 g, 1.098 g cm^−3^) was then added dropwise to afford a clear precursor solution. This precursor solution was aerosolized using a nebulizer with nitrogen as the carrier gas (2 L min^−1^) and directly injected into a tube furnace at 400 °C, and the dry nanoparticulate product was collected using a single-layer plate and frame filter press. To remove CTAB, the collected sample was placed in a quartz boat and annealed at 550 °C for 6 h under air (80 mL min^−1^) in a horizontal tube furnace to afford a white product. Hereinafter, this product will be referred to as TiO_2_.

Niobium-doped TiO_2_ nanoparticles (Nb-TiO_2_) were prepared by a similar procedure with the addition of niobium(v) ethoxide (1 mL) alongside TTIP, end up with a white product.

#### 2.1.2. Preparation of platinum catalyst on TiO_2_ and Nb-TiO_2_ nanoparticles

A hydrothermal method involving ethylene glycol was used to deposit platinum nanoparticles onto the supports, as previously described by Chen *et al.* Chloroplatinic acid hexahydrate (8 wt% in H_2_O) was used as the platinum source.^27^ Each as-prepared support (5.85 mg) was suspended in ethylene glycol (13 mL) with the aid of ultrasonication, and the H_2_PtCl_6_ solution (0.05 mL) was then added dropwise under constant stirring, followed by the addition of 1 M sodium hydroxide to adjust the pH to 9.0–10.0. The resulting mixture was agitated for 30 min to ensure adequate platinum diffusion into the surface layer of the support. The suspension was then transferred to the stainless-steel outer vessel of a 25 mL Teflon-lined autoclave reactor, which was held at 180 °C for 0.5 h before being allowed to cool to ambient temperature. The final catalyst products (denoted Pt/TiO_2_ and Pt/Nb-TiO_2_) were obtained by centrifugation at 15 000 rpm, repeated washing with deionized water, and overnight (>8 h) drying at 80 °C in a vacuum oven. With the exception of reaction time, the samples such as Pt/Nb-TiO_2_-10 and Pt/Nb-TiO_2_-40 were prepared in an identical manner.

### 2.2. Material characterization

The crystal structure and morphology of the nanoparticles were also examined by X-ray diffraction (XRD; D8 Advance, Bruker AXS, Germany) and transmission electron microscopy (TEM; Talos F200X, FEI, USA). The specific surface area was determined by the Brunauer–Emmett–Teller (BET) method and the pore structure was calculated by the Barrett–Joyner–Halenda (BJH) method using a surface analytical instrument (ASAP 2460, Micromeritics, USA) for N_2_ gas adsorption at 77 K. The surface structures were analyzed by Fourier-transform infrared (FT-IR) spectroscopy (Tensor 27, Bruker) and X-ray photoelectron spectroscopy (XPS) (ESCALAB 250Xi, Thermo Scientific). The titanium, niobium, and platinum contents were analyzed by inductively coupled plasma optical emission spectrometry (ICP-OES; 730 series, Agilent, USA). The particle size was determined using a particle size analyzer (Mastersizer 2000, Malvern, UK). The zeta potentials were recorded on a zeta potential analyzer (Zetasizer Nano ZS, Malvern, UK). The electrical conductivity of the powder samples was measured by the standard four-probe technique using a resistivity tester (FM100GH, YAOS, China) at 1 MPa. Electron paramagnetic resonance (EPR) spectra were recorded at 300 K using an EPR spectrometer (A300, Bruker).

### 2.3. Electrochemical characterization

#### 2.3.1. Preparation of catalyst inks and electrodes

For ORR activity, the ink could be obtained by blending 5 mg of catalyst, 50 μL of 5 wt% Nafion and 1 mL of ethanol under ultrasound for 30 minutes. Prior to loading the catalyst, a glassy carbon electrode (5 mm diameter, geometric area = 0.196 cm^2^) was repeatedly polished with alumina slurries (1.0 and 0.05 μm particle diameters) then ultrasonicated in water and ethanol to remove adsorbed alumina particles. A drop-coating approach was used to load the catalyst ink (16 μL) onto the electrode surface, which was then dried at room temperature to afford a catalyst film. Commercial Pt/C (HESEN, Shanghai, 20 wt%) was also tested under identical conditions for comparison.

#### 2.3.2. Electrochemical measurements

Electrochemical analysis was conducted on a Gamry electrochemical analyzer with a rotating speed controller. The tests were performed using a three-electrode setup with the catalyst-coated glassy carbon working electrode, a platinum wire counter electrode, and a mercury/mercuric oxide reference electrode [0.877 V *vs.* reversible hydrogen electrode (RHE)].

Cyclic voltammetry (CV) curves were recorded from +0.2 to −0.8 V (*vs.* RHE) in O_2_-saturated 0.1 M KOH solution at a scan rate of 50 mV s^−1^. The rotating disk electrode approach was used to acquire ORR polarization curves by linear sweep voltammetry (LSV) from +0.2 to −0.8 V in O_2_-saturated 0.1 M KOH solution at a scan rate of 10 mV s^−1^ and rotational speeds 1600 rpm. All current densities were normalized to the geometric surface area of the electrode.

The electrochemical surface area (ECSA) of platinum was calculated using the equation

1 ECSA = *Q*_H_/*mc*

where *Q*_H_ is the charge for hydrogen desorption (mC cm^−2^), *m* is the platinum loading (mg cm^−2^) in the electrode, and *c* is the charge required for the monolayer adsorption of hydrogen on a platinum surface (0.21 mC cm^−2^).

The kinetic current was calculated from the ORR polarization curves by the Koutecký–Levich equation:2
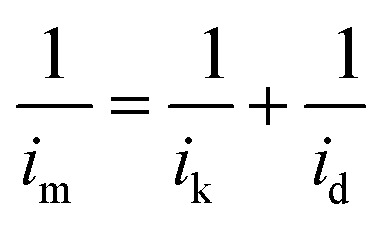
where *i*_m_ is the measured current, *i*_k_ is the kinetic current, and *i*_d_ is the diffusion-limited current. To calculate the mass activity and specific activity, *i*_k_ was standardized by the platinum loading amount and the ECSA of the catalyst.

## 3. Results and discussion

**Fig. 1:**
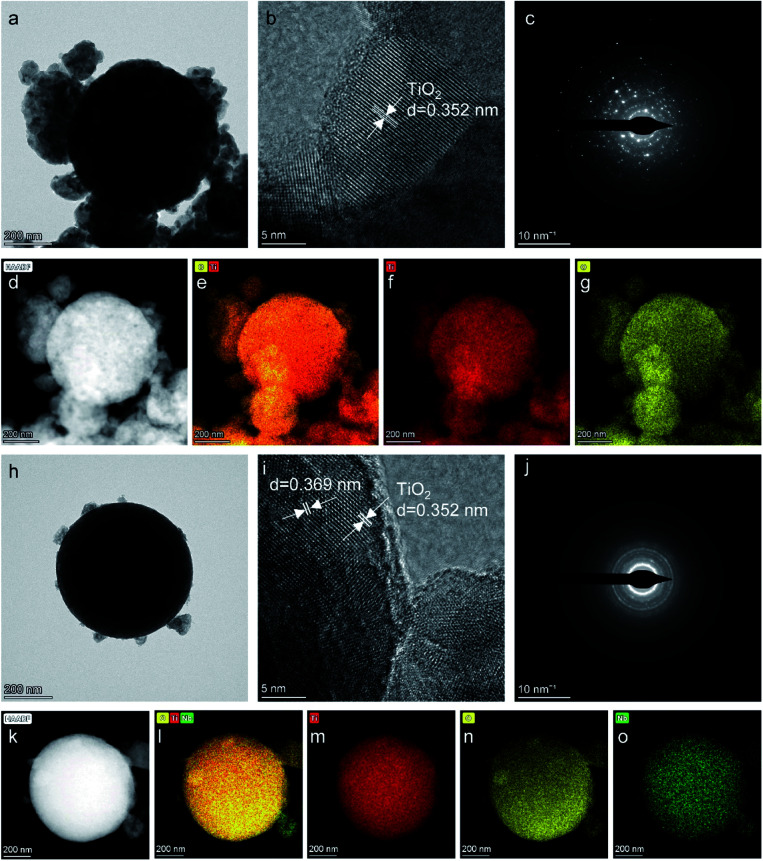
(a) Low-magnification and (b) high-resolution TEM images of TiO_2_. (c) SAED pattern of TiO_2_. (d)–(g) HAADF-STEM image of TiO_2_ and corresponding EDS elemental mapping images for titanium, oxygen, and an overlay of the two. (h) Low-magnification and (i) high-resolution TEM images of Nb-TiO_2_. (j) SAED pattern of Nb-TiO_2_. (k)–(o) HAADF-STEM image of Nb-TiO_2_ and corresponding EDS elemental mapping images for titanium, oxygen, niobium, and an overlay of the three.

The morphologies of the TiO_2_ and Nb-TiO_2_ supports were first examined by TEM, as shown in Fig. 1(a) and (h), respectively. Both supports were composed of spherical particles (200–400 nm) alongside small amounts of irregular TiO_2_. This was in accordance with the results of dynamic light scattering, which revealed *Z*-average values of 369 nm for TiO_2_ and 247 nm for Nb-TiO_2_. The high-resolution TEM image presented in Fig. 1(b) indicates the presence of highly stacked TiO_2_ layers, which displayed a spotty selected-area electron diffraction (SAED) pattern as shown in Fig. 1(c), demonstrating that single-crystalline TiO_2_ was obtained as expected. The lattice fringes with a spacing of 0.352 nm for both samples corresponded to the TiO_2_ (101) crystal plane. The EDS elemental mapping images shown in Fig. 1(d)–(g) and (k)–(o) revealed homogeneous distributions of titanium, oxygen, and niobium (in the case of Nb-TiO_2_) across the TiO_2_ nanoparticles. In Fig. 1(i) and (j), a high-resolution TEM image of Nb-TiO_2_ and a corresponding SAED pattern are presented. The SAED pattern contained diffuse rings corresponding to amorphous phases, whereas Nb-TiO_2_ was produced from amorphous TiO_2_. No lattice spacings corresponding to the Nb_2_O_5_ crystal plane at 0.375 nm were detected, and the observed lattice spacing of 0.369 nm indicated the successful doping of TiO_2_ with niobium.

**Fig. 2:**
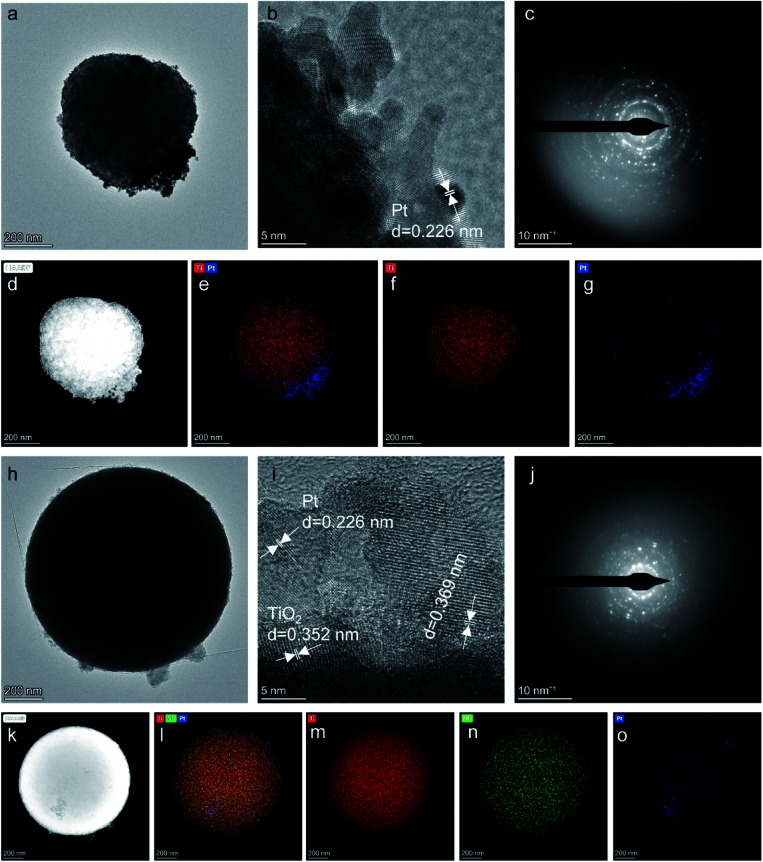
(a) Low-magnification and (b) high-resolution TEM images of Pt/TiO_2_. (c) SAED pattern of Pt/TiO_2_. (d)–(g) HAADF-STEM image of Pt/TiO_2_ and corresponding EDS elemental mapping images for titanium, platinum, and an overlay of the two. (h) Low-magnification and (i) high-resolution TEM images of Pt/Nb-TiO_2_. (j) SAED pattern of Pt/Nb-TiO_2_. (k)–(o) HAADF-STEM image of Pt/Nb-TiO_2_ and corresponding EDS elemental mapping images for titanium, niobium, platinum, and an overlay of the three.

**Fig. 3:**
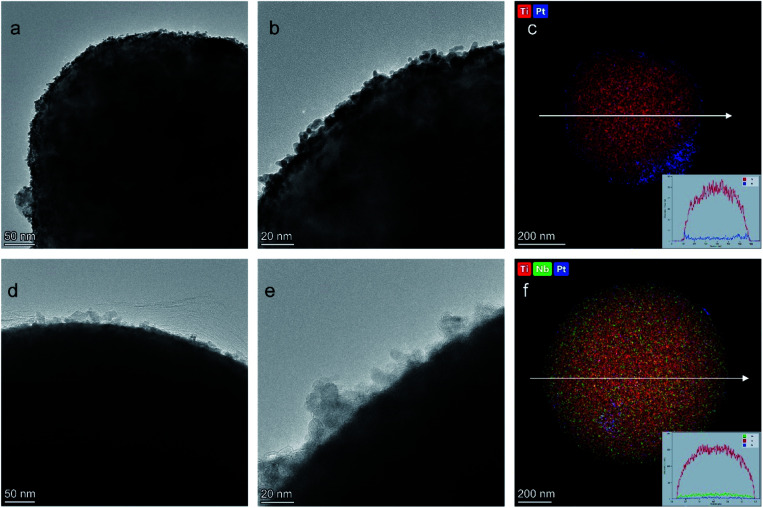
(a and b) High-resolution TEM and (c) EDS line scanning profiles images of Pt/TiO__2__. (d and e) High-resolution TEM and (f) EDS line scanning profiles images of Pt/Nb-TiO__2__.

TEM images of the as-prepared Pt/TiO_2_ and Pt/Nb-TiO_2_ are shown in Fig. 2(a) and (h) to explore the influence of the morphology on the electrochemical performance. Fig. 2(g) and (o) reveal that each support was coated with a thin platinum layer, confirming that the platinum nanoparticles were evenly distributed across the support surface. This dispersion indicates good contact between the catalyst and support, allowing the supported catalyst to exhibit good electronic conductivity.^28^ This layer structure of the platinum nanoparticles was further demonstrated by the EDS line scanning profiles, as shown in Fig. 3. Furthermore, the elemental compositions were determined by ICP-OES, as summarized in Table 1. The high-resolution TEM images and corresponding SAED patterns are presented in Fig. 2(b), (c), (i), and (j), which confirmed the presence of coexisting crystalline phases. In addition to the TiO_2_(101) crystal plane, the lattice fringes with a spacing of 0.226 nm corresponded to platinum, confirming the successful anchoring of this element on the TiO_2_ support. The EDS mapping results revealed that titanium, oxygen, and niobium were homogeneously dispersed as shown in Fig. 2(d)–(f) and (k)–(n), whereas some aggregation was observed for platinum on Pt/TiO_2_ as shown in Fig. 2(g). In the case of Pt/Nb-TiO_2_, the aggregation of the platinum nanoparticles was limited and they were well attached to the support, as observed in Fig. 2(o).

**Table 1:** Surface contents of Ti, Nb, and Pt species for the samples according to ICP-OES analysis

Sample	Elemental content (wt%)		
	Ti	Nb	Pt
TiO_2_	58.04	—	—
Nb-TiO_2_	38.96	18.39	—
Pt/TiO_2_	40.16	—	22.77
Pt/Nb-TiO_2_	31.36	14.19	21.99

**Fig. 4:**
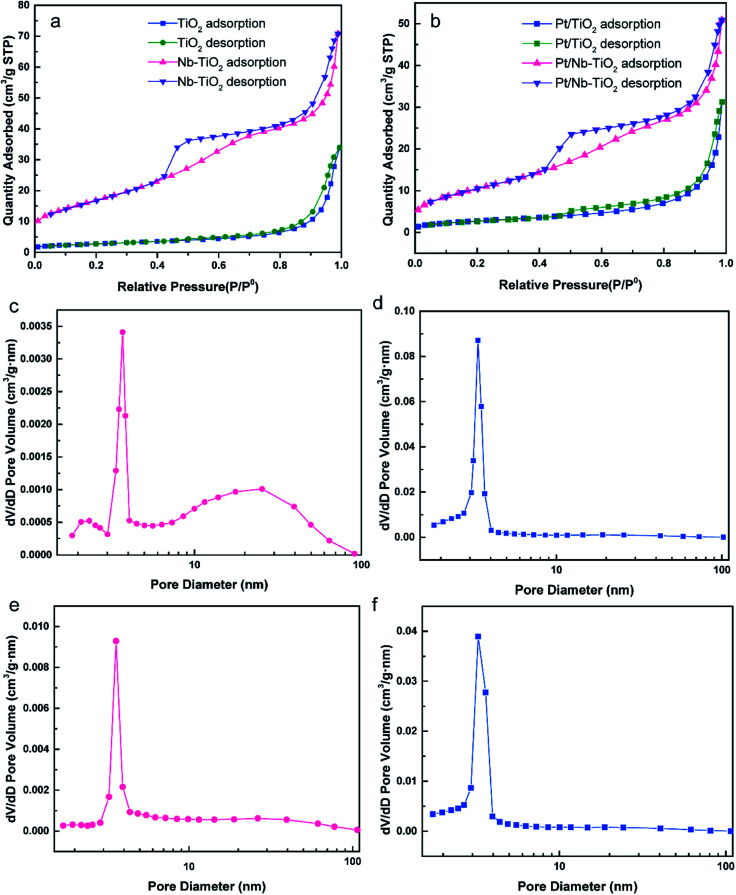
(a) N_2_ adsorption/desorption isotherms for TiO_2_ and Nb-TiO_2_. (b) N_2_ adsorption/desorption isotherms for Pt/TiO_2_ and Pt/Nb-TiO_2_. (c)–(f) Pore size distributions for (c) TiO_2_, (d) Nb-TiO_2_, (e) Pt/TiO_2_, and (f) Pt/Nb-TiO_2_.

Fig. 4 presents the nitrogen adsorption/desorption isotherms and pore size distribution curves calculated by the BJH technique for TiO_2_, Nb-TiO_2_, Pt/TiO_2_, and Pt/Nb-TiO_2_. According to the IUPAC classification scheme, the isotherm for TiO_2_, as shown in Fig. 4(a), was a combination of type-II and type-IV curves and was comparable to the isotherms previously reported for metallic aerogels.^29^ In contrast, the isotherm for Nb-TiO_2_ was a type-IV curve, featuring an apparent hysteresis loop with strong adsorption and desorption branches (capillary condensation step) at intermediate relative pressure, which can be ascribed to the presence of mesopores in this sample. The H2 hysteresis loop indicated the presence of ink-bottle-like pores with narrow necks and broader bodies, while also demonstrating the presence of mesopores (2–50 nm). This is supported by the pore size distribution curve, which indicated that the pore size for the Nb-TiO_2_ support was centered at 6.48 nm, as shown in Fig. 4(d) and Table 2. The distribution of the pore diameter of TiO_2_ at 21.04 differed from Nb-TiO_2_ in Fig. 4(d) and Table 2. The detailed pore and surface area characteristics for all samples are reported in Table 2. These findings suggest that niobium doping favors the production of homogeneous mesopores while inhibiting the formation of larger mesopores, which is in accordance with the isotherm results. The specific surface area and pore volume increased upon niobium incorporation, with the former displaying values of 9.65 and 60.00 m^2^ g^−1^ for TiO_2_ and Nb-TiO_2_, respectively.

**Table 2:** Surface area, average pore diameter, and total pore volume for TiO_2_, Nb-TiO_2_, Pt/TiO_2_, and Pt/Nb-TiO_2_

Sample	Surface area (m^2^ g^−1^)	Pore diameter (nm)	Pore volume (cm^3^ g^−1^)
TiO_2_	9.65	21.04	0.052
Nb-TiO_2_	60.00	6.48	0.11
Pt/TiO_2_	9.54	18.44	0.048
Pt/Nb-TiO_2_	37.52	7.80	0.078

Fig. 4(b), (e), and (f) show the corresponding nitrogen adsorption/desorption isotherms and pore size distribution curves for Pt/TiO_2_ and Pt/Nb-TiO_2_. The isotherms displayed a similar trend to those of the samples prior to loading with platinum, although the specific surface area of the undoped TiO_2_ remained essentially unaltered upon platinum loading whereas that of Nb-TiO_2_ decreased dramatically, indicating that the platinum entered the Nb-TiO_2_ channels to afford a large number of active sites. The minimal change in the pore size distribution suggested that the porous structure was not markedly obstructed and that the platinum would be able to exhibit high electrocatalytic activity.

**Fig. 5:**
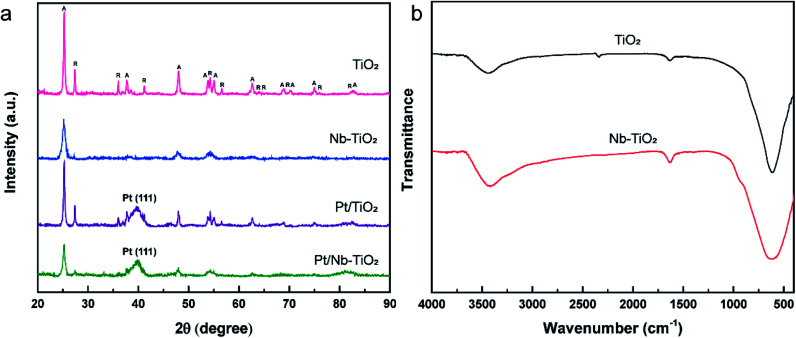
(a) XRD patterns of TiO_2_, Nb-TiO_2_, Pt/TiO_2_, and Pt/Nb-TiO_2_. The peaks labeled A and R were indexed to the anatase and rutile phases of TiO_2_, respectively. (b) FT-IR spectra of TiO_2_ and Nb-TiO_2_.

The XRD patterns of the samples were used to analyze their crystal structures, and the results are presented in Fig. 5(a). The sharp and intense diffraction peaks detected for TiO_2_ indicate a highly crystalline structure, in accordance with the sharp lattice fringes observed by TEM. The typical diffraction pattern shown in Fig. 5(a) is in good agreement with the reference data for anatase (JCPDS: 99-0008) and rutile (JCPDS: 76-0649), the two naturally occurring phases of TiO_2_. The diffraction peaks (2*θ*) at 25.30°, 37.79°, 48.04°, 53.88°, 55.06°, 62.68°, 68.75°, 70.29°, 75.04°, and 82.67° correspond to the (101), (004), (200), (105), (211), (204), (116), (220), (215), and (224) crystal planes of anatase, while those at 27.44°, 36.08°, 41.24°, 54.32°, 56.63°, 62.75°, 64.06°, 69.01°, 76.53°, and 82.35° were indexed to the (110), (101), (111), (211), (220), (002), (310), (301), (202), and (321) crystal planes of rutile, respectively.

No diffraction peaks corresponding to niobium were detected for Nb-TiO_2_, although the identification of such peaks was expected to be difficult owing to the weak crystallinity of Nb_2_O_5_. However, the observed diffraction peaks were noticeably wider than those of undoped TiO_2_, indicating the incorporation of niobium into the titania lattice to generate disordered Nb-TiO_2_. This is in accordance with the SAED pattern shown in Fig. 1(j), where niobium species were equally distributed throughout the support to afford diffuse rings corresponding to amorphous phases, while the lattice spacing of 0.369 nm observed by TEM was greater than that of 0.352 nm for pure TiO_2_, as shown in Fig. 1(i). Because the ionic radius of Nb^5+^ (64 pm) is larger than that of Ti^4+^ (60.5 pm), the lattice expansion was ascribed to the substitution of the titanium ions in TiO_2_ with niobium ions.^30^ Finally, the diffraction peaks at 39.79° for Pt/TiO_2_ and Pt/Nb-TiO_2_ were indexed to the Pt(111) crystal plane (JCPDS: 87-0646), indicating the successful loading of platinum in accordance with the EDS results shown in Fig. 3.

The FT-IR spectra of TiO_2_ and Nb-TiO_2_ are presented in Fig. 5(b). Both samples exhibited a broad absorption band at approximately 3400 cm^−1^ corresponding to the stretching vibrations of water molecules physically adsorbed on the TiO_2_ surface, in addition to an intense band at approximately 640 cm^−1^ that was ascribed to Ti–O–Ti and Ti–O stretching vibrations. The weak band at approximately 1634 cm^−1^ was assigned to the bending vibrations of the surface-bound water molecules.^31^

**Fig. 6:**
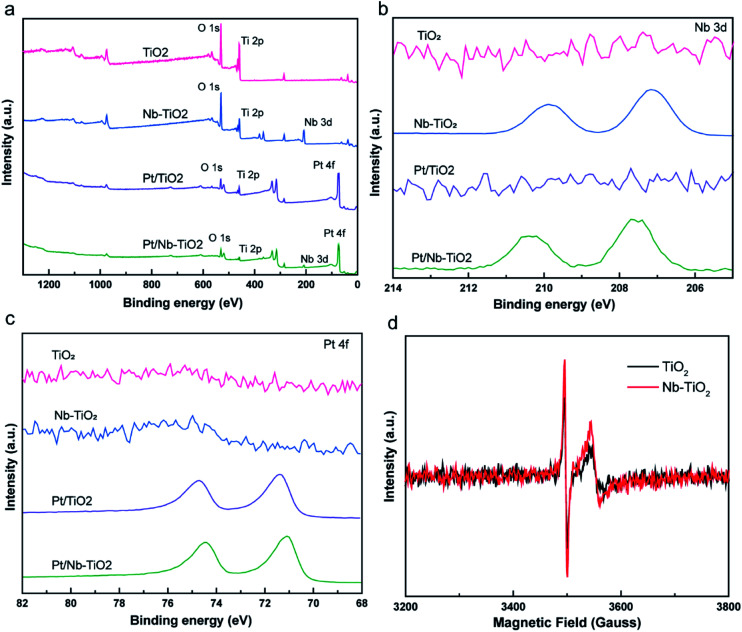
(a) XPS survey spectra, (b) niobium 3d XPS spectra, (c) platinum 4f XPS spectra for all samples and (d) EPR spectra of TiO_2_ and Nb-TiO_2_.

XPS was applied to evaluate the elemental composition and chemical states of the surface atoms in TiO_2_, Nb-TiO_2_, Pt/TiO_2_, and Pt/Nb-TiO_2_, using the carbon 1s peak at 284.8 eV as a reference. As shown in Fig. 6(a), the XPS survey spectra for TiO_2_ and Nb-TiO_2_ confirmed the presence of titanium, oxygen, and niobium (in the case of Nb-TiO_2_), while Pt/TiO_2_ and Pt/Nb-TiO_2_ displayed an additional peak corresponding to platinum, which is in good agreement with the EDS results. As shown in Fig. 6(b) and (c), narrow-scan XPS spectra further confirmed the successful doping with niobium and loading with platinum.

**Fig. 7:**
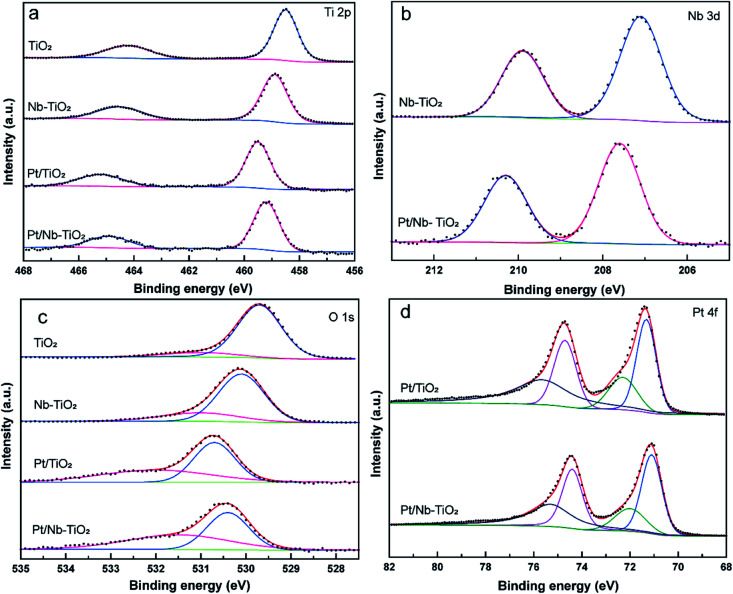
High-resolution XPS spectra of TiO_2_, Nb-TiO_2_, Pt/TiO_2_, and Pt/Nb-TiO_2_: (a) titanium 2p, (b) niobium 3d, (c) oxygen 1s, and (d) platinum 4f.

As shown in Fig. 7(a), the high-resolution titanium 2p spectrum of TiO_2_ could be deconvolved into two peaks with binding energies of approximately 464.2 and 458.5 eV and an energy separation of 5.7 eV, confirming a valence state of +4 for the titanium ions in the support.^32^ The corresponding binding energies for Nb-TiO_2_ were 464.5 and 458.9 eV, respectively. The substitution of niobium into the TiO_2_ lattice may lead to a synergistic effect with oxygen vacancies, causing an electron structural change between the conduction and valence bands. Thus, the partially replaced titanium and combined effects of niobium ions and oxygen vacancies may account for the shift of the peaks in the titanium 2p spectra toward higher binding energies. Furthermore, EPR spectroscopy was employed to confirm the presence of oxygen vacancies. As shown in Fig. 6(d), strong single-electron peaks were observed as expected.^33^ Nb-TiO_2_ afforded a characteristic EPR signal with high intensity at a *g* value of 2.003, corresponding to bridging oxygen vacancies (Ti^4+^–O–O˙).^34^ The EPR signal was slightly enhanced upon niobium incorporation.

As shown in Fig. 7(b), the high-resolution niobium 3d_5/2_ and 3d_3/2_ spectrum of Nb-TiO_2_ displayed peaks at 207.1 and 209.9 eV, which is in good agreement with previously published spectra for the Nb^5+^ valence state in niobium-doped TiO_2_.^35^ Niobium doping is known to increase electronic conductivity, which was anticipated to be advantageous for the electrochemical performance of Pt/Nb-TiO_2_ in this study. The electronic conductivity of the Nb-TiO_2_ nanoparticles was estimated to be approximately 9.85 × 10^−5^ S m^−1^ using the conventional four-probe technique, which was higher than that of the undoped TiO_2_ nanoparticles (3.48 × 10^−5^ S m^−1^), confirming that the niobium doping enhanced the conductivity. FBecause of this activation of the dispersed metal, this type of metal–support interaction can alter the electrical and catalytic properties of the catalytic centers, which is critical for an efficient electrocatalytic system. Consequently, the platinum metal in contact with the Ni–TiO_2_ support can be converted to a more active phase with enhanced charge transfer, resulting in greater catalytic activity.^36^

Deconvolution of the high-resolution oxygen 1s spectrum of TiO_2_ revealed a dominant peak at 529.7 eV and a secondary peak at 531 eV, which were assigned to Ti–O bonds and surface O–H bonds, respectively, as shown in Fig. 7(c).^37^ Furthermore, the oxygen 1s peaks of Nb-TiO_2_ shifted to higher binding energies compared with TiO_2_, indicating that the doped sample contained oxygen vacancies and niobium substitution in the TiO_2_ lattice, resulting in increased conductivity. The peaks for Pt/Nb-TiO_2_ also shifted to higher binding energies than those for pure TiO_2_, indicating strong interactions between the components of the Pt/Nb-TiO_2_ ternary system and improved electron transport.

As shown in Fig. 7(d), the high-resolution platinum 4f spectra were deconvolved into two doublets corresponding to Pt^0^ and Pt^2+^ species. The strong platinum 4f peaks were highly correlated with platinum in its zero-valent state. The Pt^0^ 4f_7/2_ binding energies were 71.32 eV for Pt/TiO_2_ and 71.1 eV for Pt/Nb-TiO_2_. This decrease in binding energy of 0.22 eV upon niobium incorporation indicates the occurrence of electron donation from the Nb-TiO_2_ to the platinum, resulting in a local increase in the electron density on platinum, *i.e.*, reduction of the platinum on the surface. This shift toward lower binding energies has also been observed in other support systems, implying that the niobium doping of TiO_2_ can alter the electronic structure of platinum atoms as a result of the SMSI effect,^38^ leading to a kinetic enhancement of the ORR due to the positive influence of the substrate.

**Fig. 8:**
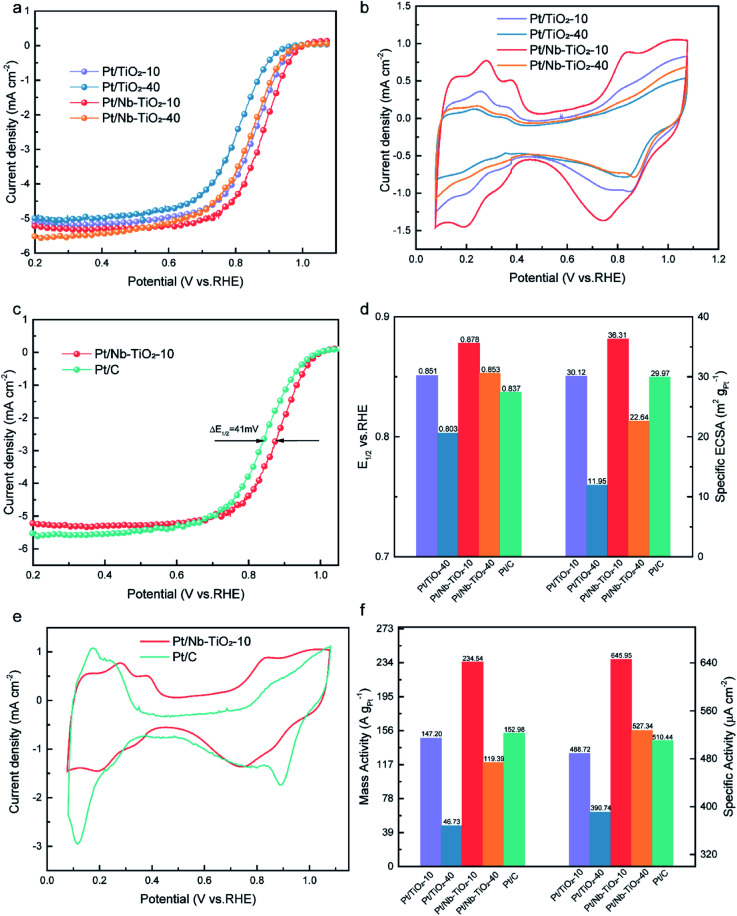
(a) ORR polarization curves and (b) CV curves for Pt/TiO_2_-10, Pt/TiO_2_-40, Pt/Nb-TiO_2_-10, and Pt/Nb-TiO_2_-40 in O_2_-saturated 0.1 M KOH electrolyte at a scan rate of 10 mV s^−1^ with a rotational speed of 1600 rpm. (c) ORR polarization curves for Pt/C and Pt/Nb-TiO_2_-10. (d) Comparison of the *E*_1/2_ and ECSA values for Pt/TiO_2_-10, Pt/TiO_2_-40, Pt/Nb-TiO_2_-10, Pt/Nb-TiO_2_-40, and Pt/C. (e) CV curves for Pt/C and Pt/Nb-TiO_2_-10. (f) Comparison of the mass activities and specific activities at 0.85 V.

To evaluate the electrocatalytic performance of the as-obtained Pt/Nb-TiO_2_ in the ORR, catalysts with and without niobium doping were prepared with different hydrothermal reaction times of 10 or 40 min, which are referred to hereinafter as Pt/TiO_2_-10, Pt/TiO_2_-40, Pt/Nb-TiO_2_-10, and Pt/Nb-TiO_2_-40. The ORR performance of the repared catalysts was investigated by CV and LSV. The ORR polarization curves were recorded in O_2_-saturated 0.1 M KOH solution with a scan rate of 10 mV s^−1^ and a rotational speed of 1600 rpm. The cyclic voltammograms were obtained at a scan rate of 50 mV s^−1^ in the same electrolyte to determine the ECSA of platinum based on hydrogen adsorption/desorption.^39^ The ORR polarization and CV curves are presented in Fig. 8(a) and (b). As demonstrated in Fig. 8(b), the hydrogen adsorption/desorption peak area in the CV curves steadily decreased with voltage cycles, indicating a reduction in the ECSA of platinum.^40^ The results indicated that Pt/Nb-TiO_2_-10 possessed the optimal electrochemical properties. Therefore, this catalyst was next compared to commercial Pt/C, as shown in Fig. 8(c) and (e). The Pt/Nb-TiO_2_-10 sample, but not Pt/C, exhibited a large Faradaic region, which may have been attributable to the SMSI of metal-oxide supported catalysts.^41^

As shown in Fig. 8(c) and (d), the half-wave potential (*E*_1/2_) of Pt/TiO_2_-10 was measured to be 0.851 V *vs.* RHE. Upon extending the hydrothermal reaction time to 40 min, *E*_1/2_ decreased to 0.803 V. The activity of the platinum nanoparticles thus decreased with increasing hydrothermal reaction time as a result of particle expansion and agglomeration. Following niobium doping, Pt/Nb-TiO_2_-10 displayed an *E*_1/2_ of 0.878 V, which surpassed that of Pt/C (0.837 V, Δ*E* = 0.41 mV). SMSI effect show that the incorporation of niobium can enhance the ORR activity, which is consistent with prior findings.^42^ In addition, the ECSA of platinum was found to be markedly greater for Pt/Nb-TiO_2_-10 (36.31 m^2^ g^−1^) than for the Pt/C catalyst (29.97 m^2^ g^−1^), which was ascribed to the small size and efficient dispersion of the platinum catalyst on the porous Nb-TiO_2_-10 support. These findings suggested that Pt/Nb-TiO_2_ would exhibit outstanding ORR activity. Indeed, as shown in Fig. 8(f), the mass activity of Pt/Nb-TiO_2_-10 (234.54 A g_Pt_^−1^ at 0.85 V) was found to greatly exceed that of Pt/C (152.98 A g_Pt_^−1^ at 0.85 V), and the former also displayed a superior specific activity (645.95 μA cm^−1^) compared with Pt/C (510.44 μA cm^−1^).

The obtained results demonstrate that niobium addition can improve the electrocatalytic efficiency of Pt/Nb-TiO_2_ catalysts because the distributed platinum, niobium, and titanium can promote the SMSI effect and act synergistically to regulate the ORR activity. Thus, the Pt/Nb-TiO_2_ catalyst outperformed a commercial Pt/C catalyst in mediating the ORR, which can be attributed to the advantages of the Pt/Nb-TiO_2_ support over conventional carbon black supports, such as the electronic structure change of platinum due to synergistic interactions with the Pt/Nb-TiO_2_ support.

## 4. Conclusions

The results described in this work demonstrate the successful application of an aerosol-assisted method to prepare a non-carbon electrocatalyst support. The obtained findings are consistent with previous research indicating that niobium doping can improve electrical conductivity and enhance the SMSI effect. When comparing our results to those of previous studies in Table 3, our work achieved the excellent mass activity. Furthermore, it is important to note that the aerosol-assisted approach has the advantages of reproducibility, simplicity, and high efficiency. These findings have some significant implications, but they also have certain limitations. Although samples prepared in this manner are resistant to agglomeration, the particle size distribution is not uniform owing to the inability of current aerosol-based technology to precisely control the diameter of each droplet. Consequently, further work is required to construct improved systems in which the aperture and particle size can be carefully regulated. Exploring the underlying mechanisms of aerosol-assisted technology to enable tuning of the structure of the target product by adjusting the composition and ratio of the precursor solution is an important direction for the future.

**Table 3:** Summary of mass activities for Nb-doped TiO_2_ as catalyst support for ORR and comparison with the present work's results

Author	Sample	ORR mass activity (A g_Pt_^−1^)
L. Chevallier^43^	Pt/Nb-TiO_2_	9.2
M. Kim^41^	Pt/Nb-TiO_2_	81
C. He^44^	Pt/aerogel-NTO	150
K. Senevirathne^45^	Pt_0.62_Pd_0.38_/Nb_0.07_Ti_0.93_O_2_	157
N. R. Elezović^46^	Pt/Nb-TiO_2_	172
This work	Pt/C	152.98
This work	Pt/Nb-TiO_2_	234.54

## Author contributions

Xin Fu: writing – original draft. Ruisong Li: investigation, conceptualization. Yucang Zhang: conceptualization, methodology, investigation, writing – original draft, writing – review & editing.

## Conflicts of interest

There are no conflicts to declare.

## Supplementary Material
